# Hospitals of the World

**Published:** 1893-03-25

**Authors:** 


### HOSPITALS OF THE WORLD.*

### THE PROGRESS OF ENGLISH NURSING (*continued*).

In 1859 the first district nurse began her work in Liverpool under the instigation of Mr. W. Rathbone, M.P. In London Mrs. Stuart Wortley acted as pioneer in nursing the sick poor in their own homes. Chiefly by her efforts the East London Nursing Society was founded in 1868. This Society was supplemented in 1874 by the Metropolitan Nursing Association in Bloomsbury Square, directed by Miss Florence Lees (Mrs. Craven). To her is largely due the zeal, ability, and tact among the nurses which have made them in the teeth of much prejudice and ignorance so great a blessing to the suffering poor in London and other towns. Other associations have since been formed for further developing this work, which received an additional stimulus by the Queen's dedication of the Women's Jubilee offering to its needs. And in 1890 the Rural Nursing Association was formed for providing the same blessing of fully trained district nurses for the country.

Since the time of the Crimean war English nurses have never been backward in volunteering for the care of the wounded in foreign countries. During the Franco-German war Miss Florence Lees went out to Metz to nurse the wounded, and had charge of the ambulance at Homburg, while Miss Byron and some of the All Saints' Sisters also went to the scene of battle. In 1875 a staff of trained nurses went to Bucharest to nurse the wounded Servians. In 1879 a staff of trained nurses was sent to the Transvaal, to attend our wounded, and a further staff of army sisters went to Pretoria on the same mission. In 1882, during the Egyptian war, and again in 1885 nursing sisters were employed in the Egyptian ports, and on board the hospital ships.

In 1883, Queen Victoria founded the Order of the Royal Red Cross, for the purpose of rewarding women who give special service to those wounded in war. The decoration consists of a red Maltese cross, bearing the words "Faith, Hope, and Charity," and is worn on the left breast.

Of equal importance as marking the assured position of the nurse was the establishment in 1887 of the Royal National Pension Fund for Nurses, of which the Princess of Wales, three years later, became president. Its work has been largely supplemented by the establishment of what has since been named from one of its largest contributors, the Junius Morgan Fund, for aiding superannuated nurses, and those incapacitated by sickness.

During the last few years a beginning has been made in the diffusion of trained nurses through the British Empire. It has been seen that India early received attention, though until the formation of Lady Dufferin's Fund in 1884 the efforts were of an isolated character. By the association formed in connection with this fund, native women are being trained as nurses, and appear to do well. Since 1885 Lady Roberts has commenced a reform in the military hospitals of India. She has organised an association of Indian Sisters, and established homes for them in the hills. The work is at present only in its infancy, yet the death-rate at Murree from typhoid fever is said to have been reduced since the introduction of the Sisters from 52 to 17 per cent.

In South Africa, nursing has been well organised from home, and at all the important hospitals satisfactory training is required or given.

In many of the colonies, however, trained nursing has hardly yet found a foothold. Of the Gold Coast, for instance, we read "at all hospitals except Accra there are only male nurses, who have absolutely no instruction or training before taking up their duties." In British Guiana the attendants are selected from applicants belonging to the general labouring population, and there are no means of specially training them, though at first they are only engaged on probation, and pick up what they can while so employed. In Australia, at the Melbourne hospital, the nurses are recruited from any class, and during the first year of service have much scrubbing to do. There is no nursing home and no lectures. Perhaps at Malta and Gozo the arrangements sound most odd. "Apparently the ordinary duties of men and women may be reversed on these islands. In reading the instructions for the guidance of the officers and servants of the charities of Malta and Gozo, it is rather embarrassing to find that the nurses are not allowed to wear beards, and of the porter it is written "she shall lock the outer gates at sunset."

* "Hospitals and Asylums of the World," 4 vols., and Portfolio of Plans. (Messrs. Churchill and the Scientific Press. 1893.)

